# Aqua­dinitrato(quioxalino[2,3-*f*][1,10]phenanthroline)nickel(II) monohydrate

**DOI:** 10.1107/S1600536809011441

**Published:** 2009-03-31

**Authors:** Jia-Tian Wang, Xin Xiao, Yun-Qian Zhang, Sai-Feng Xue, Qian-Jiang Zhu

**Affiliations:** a Key Laboratory of Macrocyclic and Supramolecular Chemistry of Guizhou Province, Guizhou University, Guiyang 550025, People’s Republic of China; b Institute of Applied Chemistry, Guizhou University, Guiyang 550025, People’s Republic of China

## Abstract

In the crystal of the title compound, [Ni(NO_3_)_2_(C_18_H_10_N_4_)(H_2_O)]·H_2_O, the Ni^II^ ion is coordinated in a distorted octahedral geometry by two N atoms of the 1,10-phenanthroline moiety of the ligand, three O atoms from two nitrate anions and an O atom from one water mol­ecule. O—H⋯O hydrogen bonds between the coordinated and the solvent water molecules and between these water molecules and the nitrate O atoms help to establish the crystal packing.

## Related literature

For transition metal complexes and their potential applications as functional materials and enzymes, see: Noro *et al.* (2000 ▶); Yaghi *et al.* (1998 ▶). For quinoxaline derivates and 1,10-phenanthroline as electron-transporting materials, see: Ambroise & Maiya (2000 ▶); Lo & Hui (2005 ▶); Thomas *et al.* (2005 ▶). 
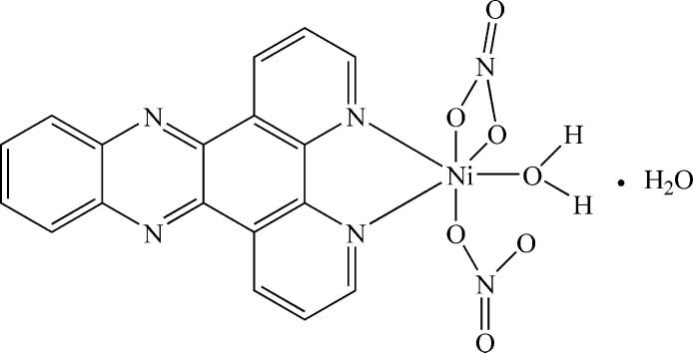


## Experimental

### 

#### Crystal data

[Ni(NO_3_)_2_(C_18_H_10_N_4_)(H_2_O)]·H_2_O
*M* *_r_* = 501.04
Monoclinic, 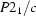

*a* = 7.300 (3) Å
*b* = 27.872 (12) Å
*c* = 9.950 (4) Å
β = 109.005 (6)°
*V* = 1914.1 (14) Å^3^
*Z* = 4
Mo *K*α radiation
μ = 1.08 mm^−1^
*T* = 293 K
0.24 × 0.21 × 0.19 mm

#### Data collection

Bruker SMART CCD area-detector diffractometer
Absorption correction: multi-scan (*SADABS*; Bruker, 2005 ▶) *T*_min_ = 0.792, *T*_max_ = 0.805
12703 measured reflections
3338 independent reflections
2255 reflections with *I* > 2σ(*I*)
*R*_int_ = 0.062

#### Refinement

*R*[*F* ^2^ > 2σ(*F* ^2^)] = 0.047
*wR*(*F* ^2^) = 0.125
*S* = 0.99
3338 reflections
314 parameters
4 restraints
H atoms treated by a mixture of independent and constrained refinement
Δρ_max_ = 1.02 e Å^−3^
Δρ_min_ = −0.35 e Å^−3^

### 

Data collection: *SMART* (Bruker, 2002 ▶); cell refinement: *SAINT* (Bruker, 2002 ▶); data reduction: *SAINT*; program(s) used to solve structure: *SHELXS97* (Sheldrick, 2008 ▶); program(s) used to refine structure: *SHELXL97* (Sheldrick, 2008 ▶); molecular graphics: *ORTEP-3 for Windows* (Farrugia, 1997 ▶); software used to prepare material for publication: *WinGX* (Farrugia, 1999 ▶).

## Supplementary Material

Crystal structure: contains datablocks global, I. DOI: 10.1107/S1600536809011441/at2754sup1.cif

Structure factors: contains datablocks I. DOI: 10.1107/S1600536809011441/at2754Isup2.hkl

Additional supplementary materials:  crystallographic information; 3D view; checkCIF report

## Figures and Tables

**Table 1:** Hydrogen-bond geometry (Å, °)

*D*—H⋯*A*	*D*—H	H⋯*A*	*D*⋯*A*	*D*—H⋯*A*
O1*W*—H1*WA*⋯O6^i^	0.83 (2)	2.02 (2)	2.839 (5)	170 (6)
O1*W*—H1*WB*⋯O2*W*^ii^	0.83 (2)	1.81 (2)	2.630 (6)	169 (6)
O2*W*—H2*WB*⋯O3^iii^	0.83 (2)	2.11 (4)	2.873 (5)	153 (7)
O2*W*—H2*WA*⋯O4	0.83 (2)	1.98 (2)	2.798 (5)	167 (6)

Symmetry codes: (i) 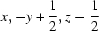; (ii) 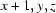; (iii) 
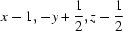
.

**Fig. 1.**
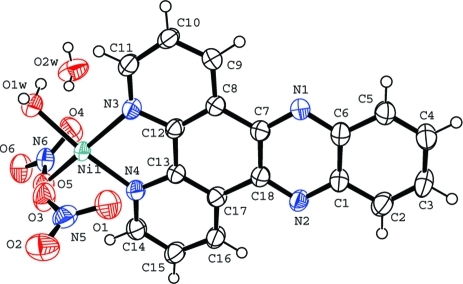
The molecular structure of (I) showing the atom-labelling scheme. Displacement ellipsoids are drawn at the 50% probability level.

## supplementary crystallographic information

### Comment

Research into transition metal complexes has been rapidly expanding because of their fascinating structural diversity, as well as their potential applications as functional materials and enzymes (Noro *et al.*, 2000; Yaghi *et al.*, 1998). And quinoxaline derivates and 1,10-phenanthroline are known to function as electron-transporting materials (Ambroise & Maiya, 2000; Lo & Hui, 2005; Thomas, *et al.*, 2005). We report here the crystal structure of the title nitrate(II) complex, (I), containing quinoxaline and 1,10-phenanthroline groups.

In the crystal of the title compound (Fig. 1), the Ni^II^ ion is coordinated by two N atoms of the 1,10-phenanthroline ligand, three O atoms from the two nitrate anions and an O atom from one water molecule. The O—H···O hydrogen bonds are observed between NO_3_^-^ and the water molecule. The hydrogen bond distances of the O1W—H1WA···O6, O1W—H1WB···O2W, O2W—H2WB···O3 and O2W—H2WA···O4, are 2.839 (5), 2.630 (6), 2.873 (5) and 2.798 (5) Å, respectively (Table 1). In the crystal structure, O—H···O hydrogen bonds interactions may help to establish the packing.

### Experimental

A solution of 1,10-phenanthroline-5,6-dione (2.1 g, 0.01 mol) in ethanol (30 ml) was added to a stirred solution of benzene-1,2-diamine (1.08 g, 0.01 mol) in ethanol (80 ml) at 293 K. The solution was stirred at room temperature for 12 h, then the 10 ml of NaOH solution (1 *M*) was added to, and the two phase mixture was well stirred for 8 min. The mixture was filtered. The residue was washed with 30 ml CH_3_CH_2_OCH_2_CH_3_. The solid product was dissolved in 90 ml ethanol, then a solution of Ni(N0_3_)_2_, (2.55 g, 0.01 mol) in H_2_O (20 ml) was added and the resulting solution was stirred for 10 min at 313 K. Then, it was left to evaporate slowly at room temperature. After two weeks, green laths and prisms of (I) were isolated.

### Refinement

Water H atoms were located in a difference Fourier map and refined freely. All other H atoms were placed in calculated positions and refined as riding, with C—H = 0.93 Å, and *U*_iso_(H) = 1.2*U*_eq_(C).

### Crystal data

**Table:**

[Ni(NO_3_)_2_(C_18_H_10_N_4_)(H_2_O)]·H_2_O	*F*(000) = 1024
*M**_r_* = 501.04	*D*_x_ = 1.739 Mg m^−^^3^
Monoclinic, *P*2_1_/*c*	Mo *K*α radiation, λ = 0.71073 Å
Hall symbol: -P 2ybc	Cell parameters from 3338 reflections
*a* = 7.300 (3) Å	θ = 1.5–25.0°
*b* = 27.872 (12) Å	µ = 1.08 mm^−^^1^
*c* = 9.950 (4) Å	*T* = 293 K
β = 109.005 (6)°	Prism, green
*V* = 1914.1 (14) Å^3^	0.24 × 0.21 × 0.19 mm
*Z* = 4	

### Data collection

**Table:**

Bruker SMART CCD area-detector diffractometer	3338 independent reflections
Radiation source: fine-focus sealed tube	2255 reflections with *I* > 2σ(*I*)
graphite	*R*_int_ = 0.062
φ and ω scans	θ_max_ = 25.0°, θ_min_ = 1.5°
Absorption correction: multi-scan (*SADABS*; Bruker, 2005)	*h* = −8→8
*T*_min_ = 0.792, *T*_max_ = 0.805	*k* = −29→33
12703 measured reflections	*l* = −11→11

### Refinement

**Table:**

Refinement on *F*^2^	Primary atom site location: structure-invariant direct methods
Least-squares matrix: full	Secondary atom site location: difference Fourier map
*R*[*F*^2^ > 2σ(*F*^2^)] = 0.047	Hydrogen site location: inferred from neighbouring sites
*wR*(*F*^2^) = 0.125	H atoms treated by a mixture of independent and constrained refinement
*S* = 0.99	*w* = 1/[σ^2^(*F*_o_^2^) + (0.0654*P*)^2^] where *P* = (*F*_o_^2^ + 2*F*_c_^2^)/3
3338 reflections	(Δ/σ)_max_ < 0.001
314 parameters	Δρ_max_ = 1.02 e Å^−^^3^
4 restraints	Δρ_min_ = −0.35 e Å^−^^3^

### Special details

**Table:**

Geometry. All e.s.d.'s (except the e.s.d. in the dihedral angle between two l.s. planes) are estimated using the full covariance matrix. The cell e.s.d.'s are taken into account individually in the estimation of e.s.d.'s in distances, angles and torsion angles; correlations between e.s.d.'s in cell parameters are only used when they are defined by crystal symmetry. An approximate (isotropic) treatment of cell e.s.d.'s is used for estimating e.s.d.'s involving l.s. planes.
Refinement. Refinement of *F*^2^ against ALL reflections. The weighted *R*-factor *wR* and goodness of fit *S* are based on *F*^2^, conventional *R*-factors *R* are based on *F*, with *F* set to zero for negative *F*^2^. The threshold expression of *F*^2^ > σ(*F*^2^) is used only for calculating *R*-factors(gt) *etc*. and is not relevant to the choice of reflections for refinement. *R*-factors based on *F*^2^ are statistically about twice as large as those based on *F*, and *R*- factors based on ALL data will be even larger.

### Fractional atomic coordinates and isotropic or equivalent isotropic displacement parameters (Å^2^)

**Table:**

	*x*	*y*	*z*	*U*_iso_*/*U*_eq_	
O1W	0.4569 (6)	0.23839 (11)	0.3617 (4)	0.0500 (8)	
O2W	−0.2513 (6)	0.25558 (15)	0.2685 (5)	0.0748 (11)	
H1WA	0.355 (5)	0.246 (2)	0.298 (5)	0.11 (2)*	
H2WA	−0.140 (4)	0.246 (2)	0.313 (6)	0.10 (2)*	
H2WB	−0.229 (10)	0.271 (2)	0.205 (5)	0.12 (3)*	
H1WB	0.559 (5)	0.2429 (19)	0.343 (6)	0.08 (2)*	
C1	0.2027 (6)	−0.07484 (15)	0.0816 (4)	0.0381 (10)	
C2	0.1687 (7)	−0.12547 (15)	0.0792 (5)	0.0491 (12)	
H2	0.1650	−0.1412	0.1607	0.059*	
C3	0.1418 (7)	−0.15036 (16)	−0.0432 (5)	0.0509 (12)	
H3	0.1195	−0.1832	−0.0441	0.061*	
C4	0.1467 (6)	−0.12769 (16)	−0.1693 (5)	0.0473 (11)	
H4	0.1275	−0.1455	−0.2518	0.057*	
C5	0.1798 (6)	−0.07968 (15)	−0.1691 (4)	0.0437 (11)	
H5	0.1829	−0.0648	−0.2521	0.052*	
C6	0.2095 (6)	−0.05199 (15)	−0.0451 (4)	0.0389 (10)	
C7	0.2656 (6)	0.02039 (14)	0.0708 (4)	0.0367 (10)	
C8	0.3059 (6)	0.07219 (14)	0.0721 (4)	0.0361 (10)	
C9	0.3115 (6)	0.09755 (15)	−0.0475 (4)	0.0435 (11)	
H9	0.2845	0.0820	−0.1345	0.052*	
C10	0.3567 (7)	0.14525 (15)	−0.0365 (4)	0.0448 (11)	
H10	0.3597	0.1624	−0.1159	0.054*	
C11	0.3984 (6)	0.16799 (15)	0.0953 (4)	0.0418 (11)	
H11	0.4364	0.2000	0.1031	0.050*	
C12	0.3390 (5)	0.09756 (13)	0.1985 (4)	0.0331 (9)	
C13	0.3222 (5)	0.07486 (14)	0.3249 (4)	0.0325 (9)	
C14	0.3228 (6)	0.08498 (15)	0.5558 (4)	0.0434 (11)	
H14	0.3396	0.1046	0.6345	0.052*	
C15	0.2730 (7)	0.03711 (15)	0.5646 (4)	0.0478 (12)	
H15	0.2554	0.0254	0.6470	0.057*	
C16	0.2501 (6)	0.00718 (15)	0.4488 (4)	0.0435 (11)	
H16	0.2178	−0.0250	0.4524	0.052*	
C17	0.2766 (6)	0.02637 (14)	0.3261 (4)	0.0343 (10)	
C18	0.2550 (6)	−0.00271 (14)	0.1970 (4)	0.0360 (10)	
N1	0.2418 (5)	−0.00420 (12)	−0.0496 (3)	0.0386 (8)	
N2	0.2264 (5)	−0.04985 (12)	0.2022 (3)	0.0393 (8)	
N3	0.3856 (5)	0.14541 (11)	0.2110 (3)	0.0364 (8)	
N4	0.3473 (5)	0.10402 (12)	0.4398 (3)	0.0371 (8)	
N5	0.8338 (6)	0.13711 (13)	0.5559 (4)	0.0462 (9)	
N6	0.1912 (5)	0.21527 (13)	0.5388 (4)	0.0448 (9)	
O1	0.7783 (5)	0.10649 (12)	0.4602 (3)	0.0641 (10)	
O2	0.9965 (4)	0.13624 (12)	0.6460 (4)	0.0659 (10)	
O3	0.7158 (4)	0.17099 (11)	0.5592 (3)	0.0546 (9)	
O4	0.0965 (5)	0.20873 (11)	0.4119 (3)	0.0591 (9)	
O5	0.3600 (4)	0.19605 (10)	0.5873 (3)	0.0455 (8)	
O6	0.1315 (5)	0.23972 (12)	0.6206 (4)	0.0602 (9)	
Ni1	0.42395 (7)	0.170749 (17)	0.40994 (5)	0.0345 (2)	

### Atomic displacement parameters (Å^2^)

**Table:**

	*U*^11^	*U*^22^	*U*^33^	*U*^12^	*U*^13^	*U*^23^
O1W	0.065 (3)	0.0339 (19)	0.049 (2)	−0.0001 (18)	0.016 (2)	−0.0016 (15)
O2W	0.057 (3)	0.073 (3)	0.090 (3)	0.008 (2)	0.018 (2)	0.035 (2)
C1	0.041 (2)	0.033 (2)	0.043 (2)	−0.0042 (19)	0.0175 (19)	−0.007 (2)
C2	0.064 (3)	0.034 (3)	0.054 (3)	−0.003 (2)	0.026 (2)	0.001 (2)
C3	0.062 (3)	0.029 (2)	0.067 (3)	−0.008 (2)	0.029 (3)	−0.011 (2)
C4	0.055 (3)	0.041 (3)	0.048 (3)	−0.006 (2)	0.020 (2)	−0.011 (2)
C5	0.052 (3)	0.037 (3)	0.042 (2)	−0.008 (2)	0.015 (2)	−0.005 (2)
C6	0.038 (2)	0.034 (3)	0.044 (2)	−0.0006 (19)	0.0121 (19)	−0.007 (2)
C7	0.041 (2)	0.033 (2)	0.035 (2)	0.0029 (18)	0.0112 (18)	−0.0014 (18)
C8	0.042 (2)	0.031 (2)	0.035 (2)	0.0029 (19)	0.0113 (18)	0.0000 (18)
C9	0.058 (3)	0.037 (3)	0.033 (2)	−0.002 (2)	0.012 (2)	−0.0036 (19)
C10	0.069 (3)	0.029 (2)	0.042 (2)	0.001 (2)	0.025 (2)	0.005 (2)
C11	0.053 (3)	0.029 (2)	0.045 (3)	−0.001 (2)	0.018 (2)	0.002 (2)
C12	0.035 (2)	0.026 (2)	0.037 (2)	0.0036 (18)	0.0107 (18)	0.0009 (18)
C13	0.034 (2)	0.029 (2)	0.034 (2)	0.0008 (18)	0.0113 (17)	−0.0007 (18)
C14	0.055 (3)	0.039 (3)	0.039 (2)	0.003 (2)	0.019 (2)	−0.005 (2)
C15	0.071 (3)	0.035 (3)	0.043 (2)	0.000 (2)	0.027 (2)	0.002 (2)
C16	0.062 (3)	0.030 (2)	0.045 (3)	−0.003 (2)	0.026 (2)	−0.003 (2)
C17	0.037 (2)	0.033 (2)	0.036 (2)	0.0023 (18)	0.0162 (18)	−0.0001 (18)
C18	0.040 (2)	0.030 (2)	0.038 (2)	0.0006 (18)	0.0131 (18)	−0.0015 (19)
N1	0.046 (2)	0.034 (2)	0.0349 (19)	−0.0001 (16)	0.0117 (16)	−0.0026 (16)
N2	0.052 (2)	0.029 (2)	0.0410 (19)	0.0008 (16)	0.0214 (17)	−0.0016 (16)
N3	0.041 (2)	0.0288 (19)	0.041 (2)	−0.0005 (15)	0.0146 (16)	−0.0008 (16)
N4	0.044 (2)	0.032 (2)	0.0355 (19)	0.0018 (16)	0.0143 (16)	−0.0030 (16)
N5	0.057 (3)	0.039 (2)	0.048 (2)	0.001 (2)	0.025 (2)	0.0066 (19)
N6	0.048 (2)	0.035 (2)	0.052 (2)	−0.0032 (18)	0.018 (2)	−0.0110 (18)
O1	0.084 (3)	0.055 (2)	0.056 (2)	0.0090 (19)	0.0272 (19)	−0.0098 (18)
O2	0.043 (2)	0.080 (3)	0.069 (2)	0.0116 (18)	0.0104 (18)	0.0144 (19)
O3	0.0478 (19)	0.0411 (19)	0.068 (2)	0.0069 (16)	0.0097 (16)	−0.0122 (16)
O4	0.057 (2)	0.064 (2)	0.0475 (19)	0.0018 (17)	0.0046 (16)	−0.0121 (16)
O5	0.0441 (18)	0.0402 (18)	0.0500 (18)	0.0038 (14)	0.0123 (14)	−0.0040 (14)
O6	0.062 (2)	0.055 (2)	0.071 (2)	0.0023 (17)	0.0325 (18)	−0.0205 (18)
Ni1	0.0469 (4)	0.0241 (3)	0.0321 (3)	−0.0013 (2)	0.0122 (2)	−0.0036 (2)

### Geometric parameters (Å, °)

**Table:**

O1W—Ni1	1.979 (3)	C11—H11	0.9300
O1W—H1WA	0.83 (2)	C12—N3	1.372 (5)
O1W—H1WB	0.83 (2)	C12—C13	1.448 (5)
O2W—H2WA	0.83 (2)	C13—N4	1.366 (5)
O2W—H2WB	0.83 (2)	C13—C17	1.393 (5)
C1—N2	1.349 (5)	C14—N4	1.334 (5)
C1—C6	1.427 (6)	C14—C15	1.393 (6)
C1—C2	1.432 (6)	C14—H14	0.9300
C2—C3	1.359 (6)	C15—C16	1.388 (6)
C2—H2	0.9300	C15—H15	0.9300
C3—C4	1.416 (6)	C16—C17	1.402 (5)
C3—H3	0.9300	C16—H16	0.9300
C4—C5	1.360 (6)	C17—C18	1.484 (5)
C4—H4	0.9300	C18—N2	1.334 (5)
C5—C6	1.411 (5)	N3—Ni1	2.033 (3)
C5—H5	0.9300	N4—Ni1	1.992 (3)
C6—N1	1.356 (5)	N5—O2	1.233 (4)
C7—N1	1.341 (5)	N5—O1	1.244 (4)
C7—C18	1.436 (5)	N5—O3	1.286 (4)
C7—C8	1.472 (5)	N6—O4	1.240 (4)
C8—C12	1.393 (5)	N6—O4	1.240 (4)
C8—C9	1.396 (5)	N6—O6	1.244 (4)
C9—C10	1.366 (6)	N6—O5	1.285 (4)
C9—H9	0.9300	O3—Ni1	2.164 (3)
C10—C11	1.399 (6)	O4—O4	0.000 (6)
C10—H10	0.9300	O5—Ni1	2.090 (3)
C11—N3	1.342 (5)		
			
Ni1—O1W—H1WA	106 (4)	N4—C14—H14	118.4
Ni1—O1W—H1WB	113 (4)	C15—C14—H14	118.4
H1WA—O1W—H1WB	116 (6)	C16—C15—C14	119.1 (4)
H2WA—O2W—H2WB	100 (6)	C16—C15—H15	120.4
N2—C1—C6	121.6 (4)	C14—C15—H15	120.4
N2—C1—C2	119.7 (4)	C15—C16—C17	118.7 (4)
C6—C1—C2	118.7 (4)	C15—C16—H16	120.7
C3—C2—C1	119.5 (4)	C17—C16—H16	120.7
C3—C2—H2	120.3	C13—C17—C16	118.6 (4)
C1—C2—H2	120.3	C13—C17—C18	118.7 (3)
C2—C3—C4	121.9 (4)	C16—C17—C18	122.6 (4)
C2—C3—H3	119.1	N2—C18—C7	121.9 (4)
C4—C3—H3	119.1	N2—C18—C17	118.6 (3)
C5—C4—C3	119.6 (4)	C7—C18—C17	119.5 (4)
C5—C4—H4	120.2	C7—N1—C6	116.5 (3)
C3—C4—H4	120.2	C18—N2—C1	116.8 (3)
C4—C5—C6	121.1 (4)	C11—N3—C12	117.5 (3)
C4—C5—H5	119.5	C11—N3—Ni1	130.3 (3)
C6—C5—H5	119.5	C12—N3—Ni1	112.2 (2)
N1—C6—C5	119.2 (4)	C14—N4—C13	117.9 (3)
N1—C6—C1	121.6 (4)	C14—N4—Ni1	128.6 (3)
C5—C6—C1	119.3 (4)	C13—N4—Ni1	113.5 (3)
N1—C7—C18	121.6 (4)	O2—N5—O1	122.7 (4)
N1—C7—C8	118.5 (4)	O2—N5—O3	119.3 (4)
C18—C7—C8	119.9 (3)	O1—N5—O3	118.0 (4)
C12—C8—C9	117.9 (4)	O4—N6—O4	0.0 (4)
C12—C8—C7	118.8 (4)	O4—N6—O6	123.3 (4)
C9—C8—C7	123.4 (4)	O4—N6—O6	123.3 (4)
C10—C9—C8	119.9 (4)	O4—N6—O5	118.0 (4)
C10—C9—H9	120.1	O4—N6—O5	118.0 (4)
C8—C9—H9	120.1	O6—N6—O5	118.7 (4)
C9—C10—C11	119.3 (4)	N5—O3—Ni1	119.9 (3)
C9—C10—H10	120.4	O4—O4—N6	0(10)
C11—C10—H10	120.4	N6—O5—Ni1	105.9 (2)
N3—C11—C10	122.5 (4)	O1W—Ni1—N4	170.98 (15)
N3—C11—H11	118.7	O1W—Ni1—N3	94.82 (14)
C10—C11—H11	118.7	N4—Ni1—N3	82.22 (13)
N3—C12—C8	122.7 (4)	O1W—Ni1—O5	87.80 (13)
N3—C12—C13	115.7 (3)	N4—Ni1—O5	92.19 (12)
C8—C12—C13	121.5 (4)	N3—Ni1—O5	160.31 (12)
N4—C13—C17	122.5 (4)	O1W—Ni1—O3	89.62 (14)
N4—C13—C12	116.2 (3)	N4—Ni1—O3	99.32 (12)
C17—C13—C12	121.3 (3)	N3—Ni1—O3	117.52 (13)
N4—C14—C15	123.1 (4)	O5—Ni1—O3	81.96 (12)

### Hydrogen-bond geometry (Å, °)

**Table:**

*D*—H···*A*	*D*—H	H···*A*	*D*···*A*	*D*—H···*A*
O1W—H1WA···O6^i^	0.83 (2)	2.02 (2)	2.839 (5)	170 (6)
O1W—H1WB···O2W^ii^	0.83 (2)	1.81 (2)	2.630 (6)	169 (6)
O2W—H2WB···O3^iii^	0.83 (2)	2.11 (4)	2.873 (5)	153 (7)
O2W—H2WA···O4	0.83 (2)	1.98 (2)	2.798 (5)	167 (6)

Symmetry codes: (i) *x*, −*y*+1/2, *z*−1/2; (ii) *x*+1, *y*, *z*; (iii) *x*−1, −*y*+1/2, *z*−1/2.
